# COMETgazing - interesting insights, lessons for clinical practice and a call for more precision using the biomarkerSCOPE

**DOI:** 10.18632/oncotarget.28698

**Published:** 2025-03-10

**Authors:** Mangesh A. Thorat

**Keywords:** DCIS, invasive breast cancer, active monitoring, overdiagnosis, TILs

It took several decades before the problem of overdiagnosis [1] in screening was widely acknowledged. And for breast cancer screening, the general perception remains that overdiagnosis is limited to excess of ductal carcinoma *in situ* (DCIS) cases. Recently published results of the COMET trial [2], which compared guideline concordant care (GCC i.e., surgery +/− adjuvant radiotherapy +/− adjuvant endocrine therapy) with active monitoring (AM) in low/intermediate grade DCIS (LG/IG-DCIS) provide interesting insights. These suggest that not an insignificant proportion of invasive cancers may also be overdiagnosed as indolent cancers with a very long lead time or cancers that spontaneously regress.

**Figure 1:**
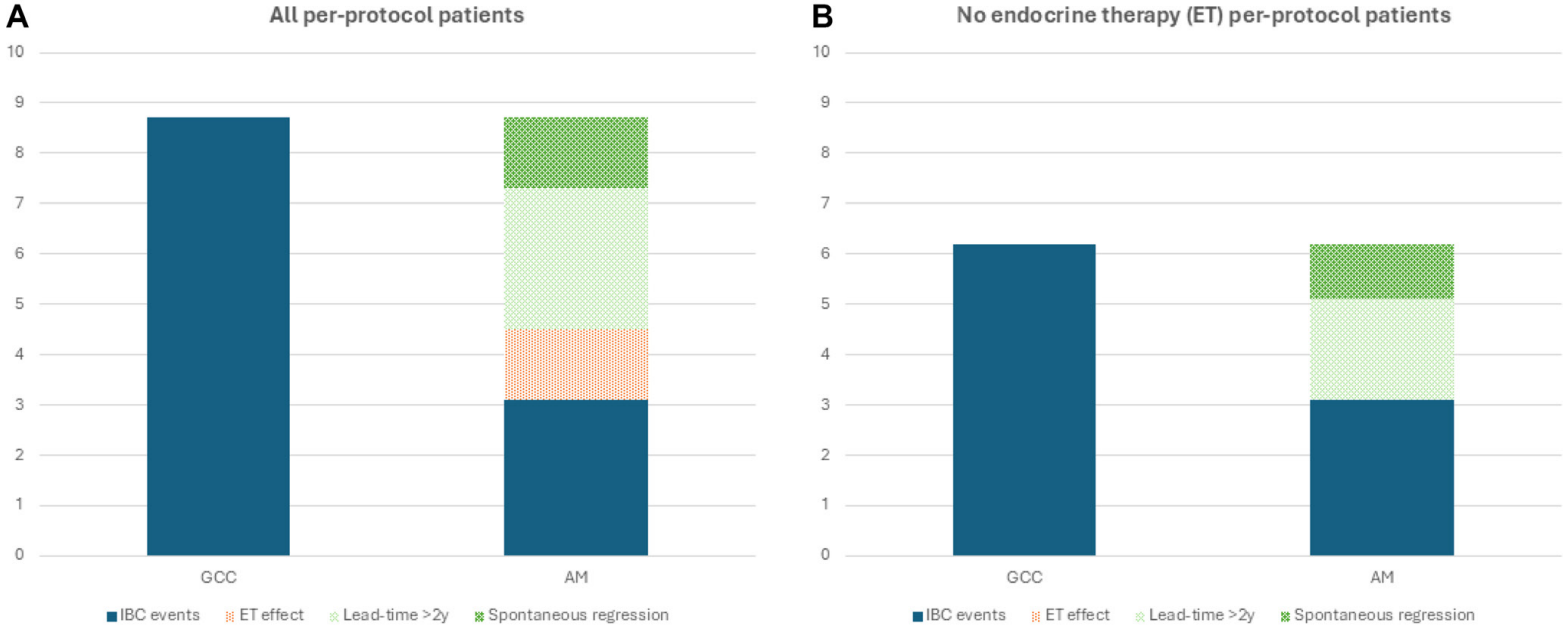
Invasive breast cancer (IBC) events in the COMET trial. (**A**) All patients in per protocol analysis; (**B**) Patients in per protocol analysis who did not receive endocrine therapy. Abbreviations: GCC: Guideline-Concordant Care; AM: Active Monitoring; ET: Endocrine Therapy; IBC: Invasive Breast Cancer.

In biopsy-proven DCIS, surgical excision has two objectives, the first is to diagnose co-existing invasive breast cancer (IBC) and the second is to reduce the risk of DCIS progressing to IBC. The median time to progression to IBC in patients allocated to no adjuvant treatment in the UK/ANZ DCIS trial [3] was 4.9 years. The current follow-up of the COMET trial [2] is quite short for progression events to occur. Therefore, a vast proportion of invasive cancers diagnosed so far in this trial are co-existing invasive cancers rather than progression of DCIS to invasive cancer. A slightly larger tumour size and a higher proportion of high-grade invasive cancers in the active monitoring arm further support this argument. The difference in the IBC events between the two trial arms therefore comprises of co-existing invasive cancers (Figure 1) not progressing to the stage of clinicoradiological detection as per the AM protocol in the trial due to (a) effect of endocrine therapy, (b) having a lead-time longer than the current follow-up of 2-years or (c) cancers undergoing spontaneous regression [4].

The 2-year cumulative rates of invasive cancer in per-protocol analyses show that this difference is 5.6%, i.e., 65% of the baseline co-existing invasive cancer rate of 8.7% in the GCC arm. Adjusting for the preventive/therapeutic efficacy of endocrine therapy used by just over 2/3rd of participants would suggest that approximately 50% of invasive cancers either have a lead-time longer than 2 years or spontaneously regress (Figure 1A). The difference (3.2%) in those who did not receive endocrine therapy against the baseline rate of 6.2% also suggests the same proportion (Figure 1B). The per protocol Kaplan-Meier plots show almost all invasive cancer diagnosis events to be clustered around follow-up visits. This suggests that these cancers do not exhibit interval symptomatic progression, a sign of more aggressive nature. Therefore, even the co-existing invasive cancers that reach a stage of clinicoradiological detection are of indolent nature. The planned long-term follow-up of the trial may shed more light on the median length of lead-time and the proportion of IBCs regressing as well as DCIS progression under different lead-time assumptions. However, what is apparent at this stage is that only a small proportion of co-existing IBCs may be worth detecting and treating in LG/IG-DCIS. Being able to distinguish between such high-risk DCIS and low-risk DCIS is a clinical and research priority.

We have shown that DCIS with multi-clonal ER expression [3, 5] has aggressive clinical behaviour like that of ER-negative DCIS. We have also shown that Cpath TILs [6] is one of the strongest predictors (HR = 3.09 in Cpath TIL-high DCIS) of progression of DCIS to IBC. The 12-year rate of progression to IBC in completely excised low/intermediate Cpath TIL-low DCIS was <4% even without adjuvant radiotherapy. Current clinical practice relies heavily on histological grade as a surrogate of biological behaviour of DCIS. Given the inherent limitations of histological grade, the use of these objective and more informative biomarkers [3, 5, 6] to identify truly low-risk DCIS needs to be explored. There are no reliable biomarkers to detect co-existing IBC. Until such tools become available, we will need to assume that non-indolent IBCs that merit detection and treatment are least likely to be associated with DCIS deemed to be low-risk by a combination of these biomarkers [3, 5, 6].

From the trial report, it is not clear what proportion of women ultimately agreed to participate in the trial. However, like the LORD trial [7], which had to change its design to an active monitoring cohort, the compliance rate of only 52% in the GCC arm makes it amply clear that a substantial proportion of such women prefer active monitoring over locoregional treatment for low-risk DCIS. To help these women exercise their informed choice in a clinically safe manner, it is incumbent upon us that we identify low-risk DCIS as accurately as possible. This would mean moving beyond grade and start using objective biomarkers that provide additional biological information [3, 5, 6].
